# Assessment of the pedal arteries with Duplex Scanning

**DOI:** 10.1590/1677-5449.200068

**Published:** 2020-11-11

**Authors:** Luciana Akemi Takahashi, Graciliano José França, Carlos Eduardo Del Valle, Luis Ricardo Coelho Ferreira

**Affiliations:** 1 Universidade Federal do Paraná – UFPR, Hospital de Clínicas – HC, Unidade Cardiopulmonar, Curitiba, PR, Brasil.; 2 Universidade Federal do Paraná – UFPR, Hospital de Clínicas – HC, Unidade de Diagnóstico por Imagem, Curitiba, PR, Brasil.

**Keywords:** Doppler ultrasonography, duplex, tibial arteries, vascular surgery procedures

## Abstract

Vascular Doppler ultrasound is a noninvasive method that can help in diagnostic and therapeutic planning in case of pedal arterial obstructive disease. The dorsalis pedis artery is the direct continuation of the anterior tibial artery and follows a straight course along the dorsum of the foot, leading medially to the first intermetatarsal space, where it gives off its terminal branches. The posterior tibial artery forks distal to the medial malleolus and gives rise to the lateral plantar and medial plantar arteries. The medial plantar artery has a smaller caliber and runs medially in the sole of the foot, while the lateral plantar artery is of larger caliber, following a lateral course in the plantar region and forming the deep plantar arch, which anastomoses with the dorsalis pedis artery via the deep plantar artery. The arteries of the foot can be assessed noninvasively with Doppler, providing an adequate level of anatomical detail.

## INTRODUCTION

Critical lower limb ischemia is the end stage of peripheral arterial disease (PAD) when arterial perfusion is insufficient to meet basic metabolic demand, characterized clinically by pain at rest or presence of trophic ulcers.^1^^,^^2^ Critical lower limb ischemia can be treated by surgical revascularization or with endovascular treatment; of which endovascular treatment is notable for its minimally invasivity and the improved options in terms of materials.^3^^,^^4^

Although use of Doppler ultrasound (vascular ultrasonography with Doppler - duplex scanning) to assess the arterial system is routine in the context of critical ischemia and PAD, adequate characterization of the pedal arteries (dorsalis pedis artery [DPA], plantar arteries, and deep plantar arch) with Doppler ultrasonography is not yet conducted routinely and assessment of these arteries is very often delegated to arteriography.^1^^,^^5^^-^7 However, now that technological advances in equipment and training of vascular ultrasonographers have yielded improvements, assessment of the pedal arteries with Doppler ultrasonography can be useful both for surgical and endovascular planning^4^^,^^8^ and for patient follow-up after treatment.^6^ The pedal arteries can also be used for retrograde arterial access via distal puncture for tibial or femoropopliteal revascularization.^9^^,^^10^ Knowledge of the ultrasonographic anatomy of the pedal arteries is indispensable for all of these applications. This review article describes the ultrasonographic anatomy and technique for assessing the pedal arteries with Doppler ultrasonography.

## ANATOMY^11^

### A. Dorsal region

The DPA is the direct continuation of the anterior tibial artery (ATA) after it passes the tibiotalar (ankle) joint. Its primary branches are the lateral tarsal and arcuate arteries. At the level of the first intermetatarsal space, the DPA gives off its terminal branches, the deep plantar artery and the first dorsal metatarsal artery.

### B. Plantar region

**Figure 1:**
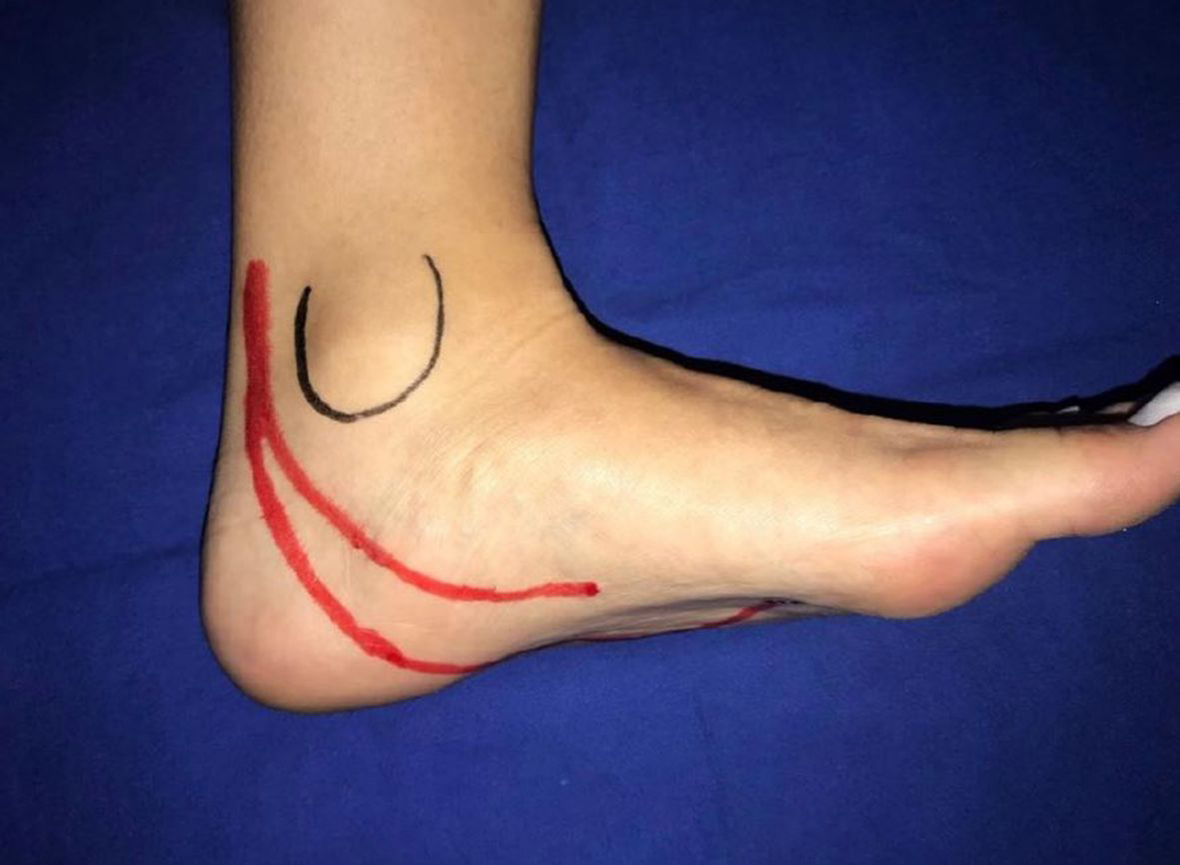
The posterior tibial artery forks distal of the medial malleolus, giving rise to the lateral plantar and medial plantar arteries.

The posterior tibial artery (PTA) forks distal of the medial malleolus, giving rise to the lateral plantar (LPA) and medial plantar (MPA) arteries (Figure 1). The MPA is of smaller caliber and runs medially along the plantar foot, whereas the LPA is of larger caliber, following a lateral path along the plantar foot, forming the deep plantar arch (DPA), which anastomoses with the DPA via the deep plantar artery, at the level of the base of the first intermetatarsal space.

### Ultrasound examination technique

The paths of the pedal arteries were marked on the skin to facilitate location. Color and spectral Doppler images of the pedal arteries were acquired in B mode using multifrequency linear transducers and a Philips Affiniti 70 ultrasound scanner (Philips Healthcare, Eindhoven, Holland).

### A. Dorsalis pedis artery

**Figure 2:**
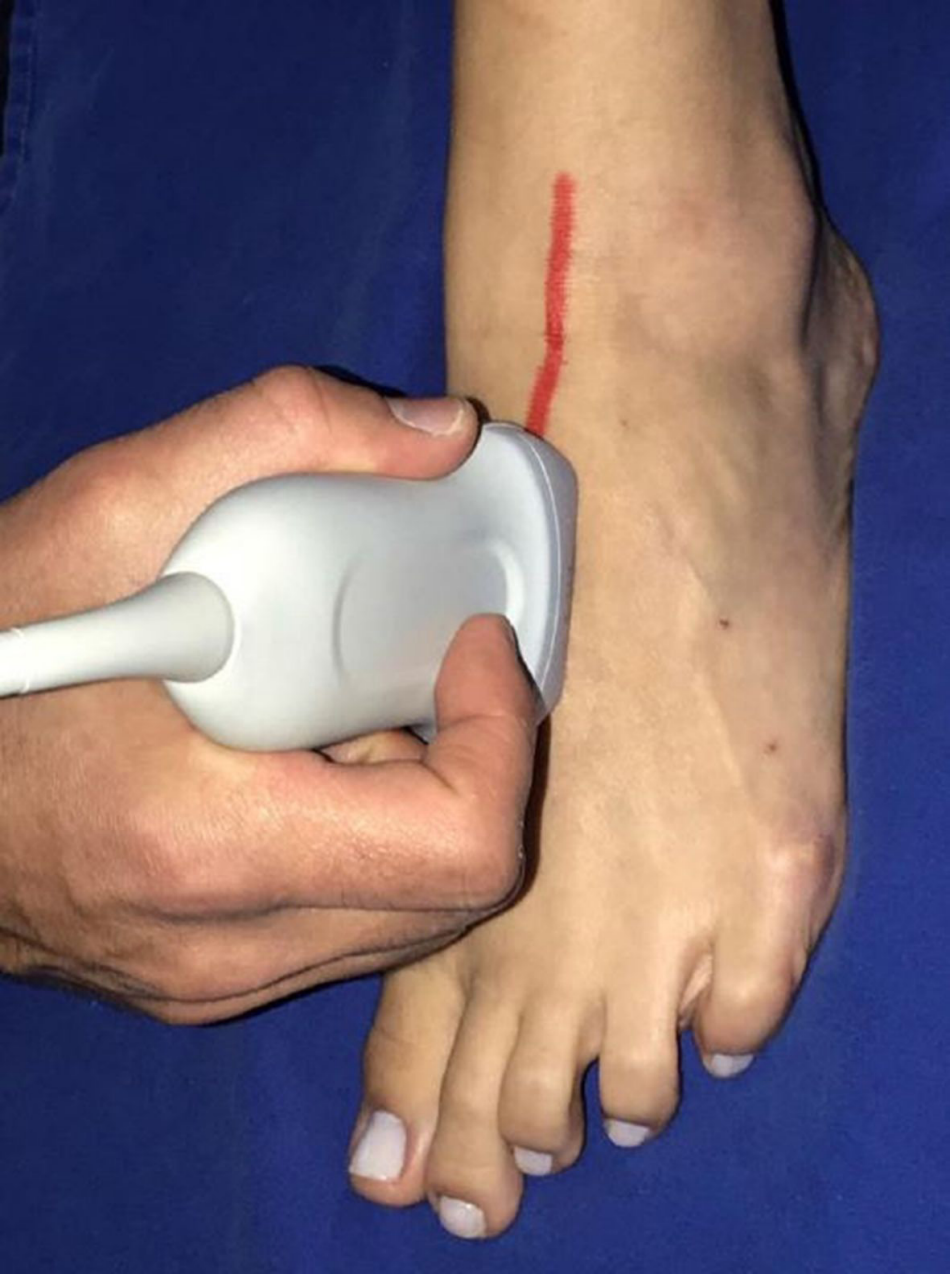
Path taken by the dorsalis pedis artery in direction of the first interdigital space.

The assessment initiates with the anterior tibial artery distal of the ankle, located anteriorly of the tibia, documenting patency and spectral curve pattern. Proceeding distally along the dorsum of the foot, the DPA is scanned, adjusting transducer frequency and depth, because the DPA is of small caliber and very superficial. The DPA follows a straight path along the dorsum of the foot, running medially to the first intermetatarsal space, where it gives rise to its terminal branches. Its topographical anatomy should be imagined as running in the direction of the first interdigital space (Figure 2).

### B. Plantar arteries and deep plantar arch

**Figure 3:**
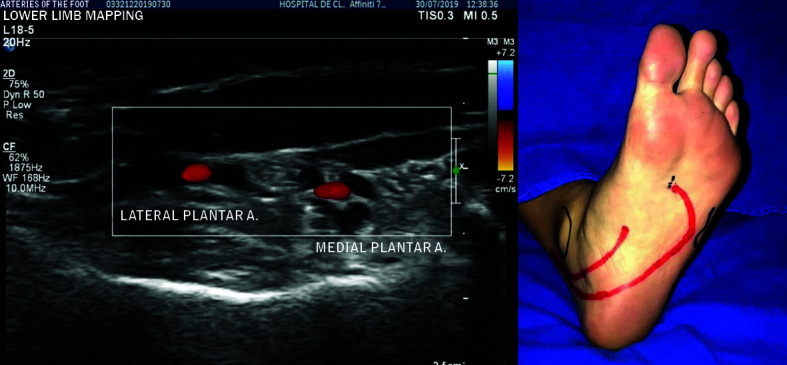
Cross-sectional images in the region of the medial plantar and lateral plantar arteries.

Next, the PTA is identified at the level of the medial malleolus, documenting patency and spectral curve pattern. Using cross-sectional images of the PTA and proceeding distally to the medial margin of the foot, the bifurcation of the PTA at the MPA and LPA is observed (Figure 3).

**Figure 4:**
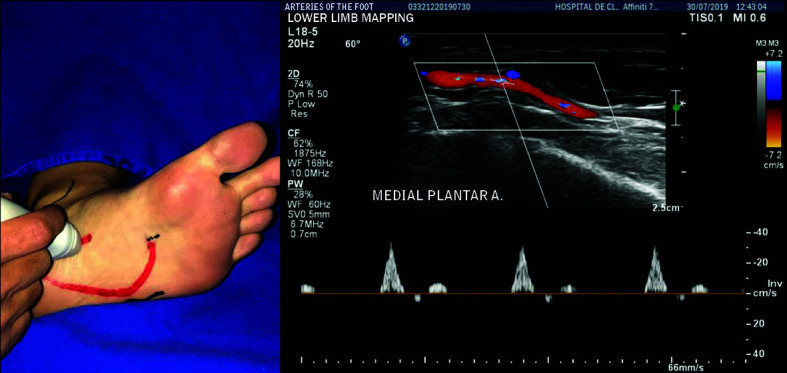
Longitudinal images of the medial plantar artery are acquired with the transducer pointing in the direction of the great toe from the more proximal part of the foot.

The MPA is the smaller caliber branch, following a path more directly in the direction of the great toe. Longitudinal images of the MPA are obtained with the transducer pointing in the direction of the great toe from the more proximal portion of the foot (Figure 4).

**Figure 5:**
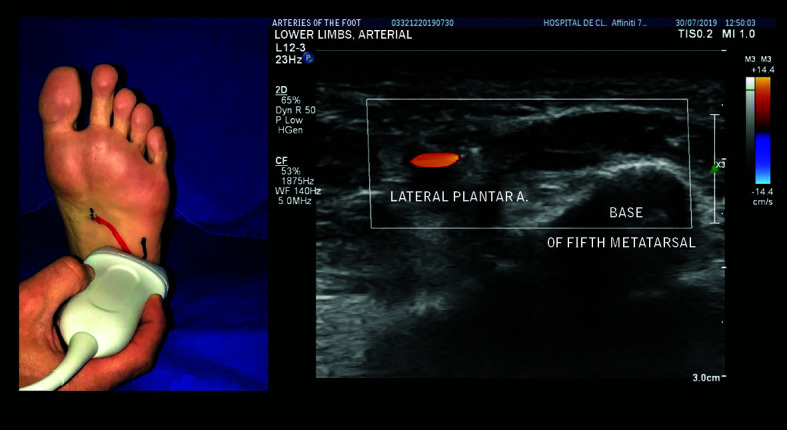
The lateral plantar artery is the lateral branch with a larger caliber than the medial plantar artery, following a path in the direction of the base of the fifth metatarsal.

**Figure 6:**
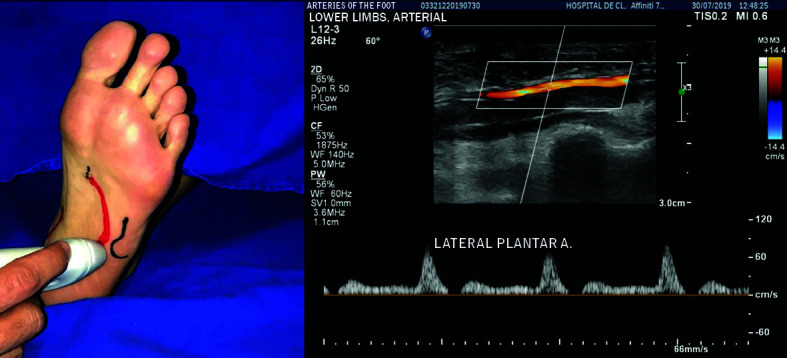
Location of lateral plantar artery, around 2.5 cm medial of the base of the fifth metatarsal.

The LPA is the larger caliber, lateral branch and follows a path in the direction of the fifth metatarsal (Figure 5). The base of the fifth metatarsal is an important landmark for assessment of the LPA because the LPA gives rise to the DPA at this point. The LPA is located about 2.5 cm medial of the base of the fifth metatarsal (Figure 6).

**Figure 7:**
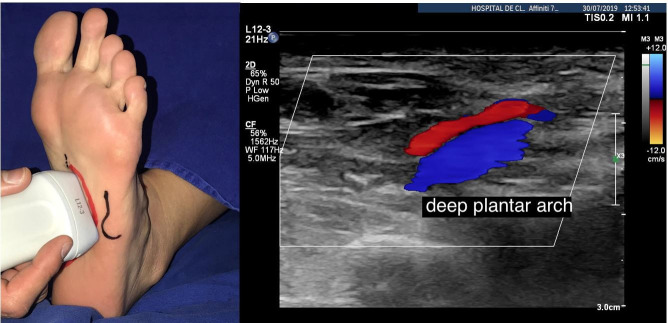
Longitudinal images of the deep plantar arch, obtained by following the lateral plantar artery and then turning the transducer so that its longer axis is in the direction of the first intermetatarsal space.

The landmark used for assessment of the DPA is once more the base of the fifth metatarsal. The LPA is identified and then the transducer is turned so that its largest axis is aligned in the direction of the first intermetatarsal space, providing longitudinal images of the DPA (Figure 7). It is important to note that, when analyzed from the plantar surface, the DPA is located deeper from the plantar fascia than the plantar lateral artery, since it runs to an anastomosis with the dorsalis pedis artery. In order to position the transducer in the direction of the first intermetatarsal space, a parallel line should be imagined joining the heads of the second to fifth metatarsals, placing the transducer against the base of the fifth metatarsal (Figure 7).

In view of all of the above, the following facts are worthy of consideration. Doppler ultrasonography is the noninvasive method of choice for assessment of the lower limb arterial system, achieving excellent correlation with arteriography (gold standard), if conducted by experienced professionals.^6^^,^^8^ However, in daily practice, Doppler ultrasonography is used much less than arteriography for assessment of the pedal arteries, possibly because of vascular ultrasonographists’ unfamiliarity with the ultrasonographic arterial anatomy of the foot. Now that the resolution of ultrasound scanners has improved, ultrasonographic characterization of the pedal arteries is possible and reproducible and, with adequate training, it does not take very long to perform.

Assessment of the plantar arch can be of prognostic value and it has been documented that a patent plantar arch is predictive of the patency of reconstructions and of healing of trophic ulcers.^12^^,^^13^ There is also a correlation between the severity of chronic renal failure (reduction in glomerular filtration rate) and plantar arch patency.^13^

It can be difficult to detect flow in the distal tibial arteries with digital arteriography in severe ischemia cases,^14^ and in such cases ultrasonography with Doppler may detect patent vessels that are suitable for revascularization, increase limb salvage rates.^7^^,^^8^^,^^15^^,^^16^ During arteriography, the pedal arteries are assessed routinely, with a lateral projection which can be used to characterize the DPA, the LPA, and the plantar arch.

This assessment can also be performed using Doppler ultrasonography, rapidly and noninvasively, both for diagnosis and for follow-up after treatment. It is concluded that the pedal arteries can be noninvasively assessed with Doppler ultrasonography, affording an excellent level of anatomic detail.
