# Supplementary material for: Assessment of the pedal arteries with Duplex Scanning
Source: J Vasc Bras. 2020 Nov 11;19:e20200068. doi: 10.1590/1677-5449.200068 (PMC8218015; doi:10.1590/1677-5449.200068)
Supplement: Translation

# Translation: Avaliação das artérias podais ao eco-Doppler

**Authors:** Luciana Akemi Takahashi, Graciliano José França, Carlos Eduardo Del Valle, Luis Ricardo Coelho Ferreira

**Affiliations:** 1 Universidade Federal do Paraná – UFPR, Hospital de Clínicas – HC, Unidade Cardiopulmonar, Curitiba, PR, Brasil.; 2 Universidade Federal do Paraná – UFPR, Hospital de Clínicas – HC, Unidade de Diagnóstico por Imagem, Curitiba, PR, Brasil.

**Keywords:** ultrassonografia Doppler, artérias da tíbia, procedimentos cirúrgicos vasculares

## Abstract

A ultrassonografia vascular com Doppler é um método não invasivo útil no diagnóstico e planejamento terapêutico da doença oclusiva das artérias podais. A artéria pediosa dorsal é a continuação direta da artéria tibial anterior e tem trajeto retilíneo no dorso do pé, dirigindo-se medialmente ao primeiro espaço intermetatarsiano, onde dá origem a seus ramos terminais. A artéria tibial posterior distalmente ao maléolo medial se bifurca e dá origem às artérias plantar lateral e plantar medial. A plantar medial apresenta menor calibre e segue medialmente na planta do pé, enquanto a plantar lateral é mais calibrosa, seguindo um curso lateral na região plantar e formando o arco plantar profundo, o qual se anastomosa com a artéria pediosa dorsal através da artéria plantar profunda. A avaliação das artérias podais pode ser realizada de maneira não invasiva com exame de eco-Doppler, com adequado nível de detalhamento anatômico.

## INTRODUÇÃO

A isquemia crítica de membro inferior é o estágio final da doença arterial periférica (DAP), quando a perfusão arterial não é suficiente para atender às demandas metabólicas basais, sendo caracterizada clinicamente por dor ao repouso ou presença de lesão trófica^1^^,^^2^. O tratamento da isquemia crítica do membro inferior pode ser realizado tanto por revascularização cirúrgica quanto por tratamento endovascular, com o tratamento endovascular se destacando por ser minimamente invasivo e com melhora das opções de materiais^3^^,^^4^.

Apesar do uso rotineiro do eco-Doppler (ultrassonografia vascular com Doppler, *duplex scan*) para a avaliação do sistema arterial no contexto de isquemia crítica e DAP, a adequada caracterização das artérias podais [artéria pediosa dorsal (APD), artérias plantares e arco plantar profundo] com o eco-Doppler ainda não é realizada de forma habitual, sendo a avaliação arterial podal muitas vezes ainda delegada à arteriografia^1^^,^^5^^-^7. Porém, com os avanços tecnológicos dos equipamentos e a melhora no treinamento dos ultrassonografistas vasculares, a avaliação das artérias podais ao eco-Doppler pode ajudar tanto no planejamento cirúrgico e endovascular^4^^,^^8^ quanto no seguimento do paciente pós-tratamento^6^. As artérias podais ainda podem ser usadas como via de acesso arterial retrógrado via punção distal para recanalizações tibiais ou femoropoplíteas^9^^,^^10^. Para todas essas aplicações, é indispensável o conhecimento da anatomia ultrassonográfica das artérias podais. Neste artigo de revisão, serão descritas a anatomia ultrassonográfica e a técnica para a avaliação das artérias podais ao eco-Doppler.

## ANATOMIA^11^

### A. Região dorsal

A APD é a continuação direta da artéria tibial anterior (ATA) após cruzar a articulação tibiotalar. Tem como ramos principais as artérias tarsal lateral e arqueada. No nível do primeiro espaço intermetatarsiano, a APD dá origem a seus ramos terminais, que são a artéria plantar profunda e a artéria metatarsiana dorsal do hálux.

### B. Região plantar

A artéria tibial posterior (ATP) distalmente ao maléolo medial se bifurca e dá origem às artérias plantar lateral (APL) e plantar medial (APM) (Figura 1). A APM apresenta menor calibre e segue medialmente na planta do pé, enquanto a APL é mais calibrosa, segue um curso lateral na região plantar e forma o arco plantar profundo (APP), o qual se anastomosa com a APD através da artéria plantar profunda, no nível da base do primeiro espaço intermetatarsiano.

**Figura 1:** A artéria tibial posterior se bifurca distalmente ao maléolo medial e dá origem às artérias plantar lateral e plantar medial.

### Técnica de exame ultrassonográfico

Para facilitar a localização dos vasos, foi realizada a marcação cutânea dos trajetos vasculares arteriais podais. Foram realizadas imagens ao modo B, colorido e espectral das artérias podais utilizando transdutores lineares multifrequenciais do aparelho Philips Affiniti 70 (Philips Healthcare, Eindhoven, Holanda).

### A. Artéria pediosa dorsal

Inicia-se a avaliação pela artéria tibial anterior distal na região do tornozelo, localizada anteriormente à tíbia, documentando perviedade e padrão de curva espectral. Prossegue-se distalmente na região dorsal do pé, insonando a APD e ajustando a frequência do transdutor e a profundidade, pois a APD é de pequeno calibre e muito superficial. A APD tem trajeto retilíneo no dorso do pé, dirigindo-se medialmente ao primeiro espaço intermetatarsiano, onde dá origem a seus ramos terminais. Deve-se, na anatomia topográfica, imaginar o trajeto da APD indo em direção ao primeiro espaço interdigital (Figura 2).

**Figura 2:** Trajeto da artéria pediosa dorsal indo em direção ao primeiro espaço interdigital.

### B. Artérias plantares e arco plantar profundo

Em seguida, identifica-se a ATP na altura do maléolo medial, documentando perviedade e padrão de curva espectral. Utilizando cortes transversais da ATP, prosseguir distalmente até o rebordo medial do pé até observar a bifurcação da ATP nas APM e APL (Figura 3).

**Figura 3:** Cortes transversais na topografia das artérias plantar medial e plantar lateral.

A APM é o ramo de menor calibre, tendo trajeto com sentido apontando mais diretamente em direção ao hálux. Imagens longitudinais da APM são obtidas com o transdutor apontado na direção do hálux a partir da porção mais proximal do pé (Figura 4).

**Figura 4:** Imagens longitudinais da artéria plantar medial são obtidas com o transdutor apontado na direção do hálux a partir da porção mais proximal do pé.

A APL é o ramo lateral, mais calibroso e com trajeto em direção à base do quinto metatarso (Figura 5). A base do quinto metatarso é um importante marco topográfico para avaliação da APL, pois, nessa região, a APL dá origem ao APP. A APL está localizada cerca de 2,5 cm medialmente à base do quinto metatarso (Figura 6).

**Figura 5:** A artéria plantar lateral é o ramo lateral e mais calibroso que a plantar medial, apresentando trajeto em direção à base do quinto metatarso.

**Figura 6:** Localização da artéria plantar lateral, cerca de 2,5 cm medialmente à base do 5º metatarso.

Para a caracterização do APP, novamente deve-se utilizar a base do quinto metatarso como reparo anatômico. Identifica-se a APL, e o transdutor é, então, orientado com seu maior eixo em direção ao primeiro espaço intermetatarsiano, para se obter cortes longitudinais do APP (Figura 7). É importante notar que o APP está localizado mais profundamente em relação à fáscia plantar do que a artéria plantar lateral quando analisado a partir da face plantar, pois vai se anastomosar com a pediosa dorsal. Para posicionar o transdutor em direção ao primeiro espaço intermetatarsal, é preciso imaginar uma linha paralela unindo as cabeças do 2º ao 5º metatarsos, sendo o transdutor posicionado junto à base do 5º metatarso (Figura 7).

**Figura 7:** Corte longitudinal do arco plantar profundo, obtido seguindo-se a artéria plantar lateral e, então, orientando o transdutor com seu maior eixo em direção ao primeiro espaço intermetatarsiano.

Tendo em vista o que foi exposto, podem-se ponderar os seguintes fatos. O eco-Doppler é o método não invasivo de escolha para a avaliação do sistema arterial dos membros inferiores, obtendo-se excelente correlação com a arteriografia (padrão-ouro), se realizado por profissionais experientes^6^^,^^7^. Porém, na prática diária, o eco-Doppler apresenta-se muito aquém à arteriografia na avaliação das artérias podais, talvez pela não familiaridade do ultrassonografista vascular com a anatomia ultrassonográfica arterial do pé. Com a melhora de resolução dos aparelhos de ultrassonografia, a caracterização ultrassonográfica das artérias podais é factível e reprodutível e, com treinamento adequado, não demanda muito tempo para a realização.

A avaliação do arco plantar pode ter valor prognóstico, já tendo sido documentado que a presença de um arco plantar pérvio é preditivo de perviedade de reconstruções e cicatrizações em lesões tróficas^12^^,^^13^. Existe também uma correlação entre a gravidade de insuficiência renal crônica (queda na taxa de filtração glomerular) e a perviedade do arco plantar^13^.

Em casos de isquemia grave, a arteriografia digital pode ter dificuldade de detectar fluxo nas artérias tibiais distais^14^, sendo que, nesses casos, o ultrassonografia com Doppler pode revelar vasos pérvios que se prestam a revascularização, com aumento das taxas de salvamento de membro^7^^,^^8^^,^^15^^,^^16^. Na arteriografia é feita rotineiramente a avaliação das artérias podais, com incidência em perfil do pé, em que é possível caracterizar a APD, a APL e o arco plantar.

Com a utilização do eco-Doppler, pode-se realizar essa avaliação de forma rápida e não invasiva, tanto para diagnóstico quanto para seguimento pós-tratamento. Conclui-se que a avaliação das artérias podais pode ser realizada de maneira não invasiva com exame de eco-Doppler, com excelente nível de detalhamento anatômico.
